# Employment status and desire for work in severe mental illness: results from an observational, cross-sectional study

**DOI:** 10.1007/s00127-021-02088-8

**Published:** 2021-04-16

**Authors:** Uta Gühne, Alexander Pabst, Margrit Löbner, Johanna Breilmann, Alkomiet Hasan, Peter Falkai, Reinhold Kilian, Andreas Allgöwer, Klemens Ajayi, Jessica Baumgärtner, Peter Brieger, Karel Frasch, Stephan Heres, Markus Jäger, Andreas Küthmann, Albert Putzhammer, Bertram Schneeweiß, Michael Schwarz, Thomas Becker, Markus Kösters, Steffi G. Riedel-Heller

**Affiliations:** 1 Medical Faculty, Institute of Social Medicine, Occupational Health and Public Health (ISAP), University of Leipzig, Philipp-Rosenthal-Straße 55, 04103 Leipzig, Germany; 2 Department of Psychiatry II, Ulm University, BKH Günzburg, Günzburg, Germany; 3 Department of Psychiatry, Psychotherapy and Psychosomatic, Medical Faculty, University of Augsburg, BKH Augsburg, Augsburg, Germany; 4 Department of Psychiatry and Psychotherapy, University Hospital Munich, Munich, Germany; 5 Institute for Epidemiology and Medical Biometry, Ulm University, Ulm, Germany; 6 Kbo-Isar-Amper Hospital, Munich, Germany; 7 District Hospital Donauwörth, Donauwörth, Germany; 8 District Hospital Kempten, Kempten, Germany; 9 District Hospital Memmingen, Memmingen, Germany; 10 District Hospital Kaufbeuren, Kaufbeuren, Germany

**Keywords:** Employment status, Desire to work, Supported employment, Predictors, Work ability

## Abstract

**Purpose:**

People with a severe mental illness (SMI) are at particular risk of occupational exclusion. Among the approaches to occupational rehabilitation, supported employment (SE) has been proven to be the most effective. A requirement to enter SE-programs is that individuals must want to seek competitive employment. The aim of this work is to investigate the relationship between serious mental illness and the desire to work including potential predictors.

**Methods:**

This is a cross-sectional observational study of patients with SMI aged 18–65 years (*n* = 397). Patients were interviewed by trained staff using standardised instruments. The relationship between potential predictors and a strong preference for employment were analysed using a hierarchic binary logistic regression model.

**Results:**

Only about one-quarter (27.9%) of SMI patients is in competitive employment. Another quarter is unemployed (25.9%). Results show that the desire for competitive employment is strong among more than half of the SMI patients. Among the unemployed, two-thirds express a strong desire for work. These individuals are an ideal target group for SE interventions. Comorbid chronic physical illness, diagnosis, and the subjectively judged ability to work are associated with the desire for work.

**Conclusion:**

Our data confirm a substantial exclusion of individuals with SMI from the workforce. In general, care needs for workplace interventions are not being met and leave much room for improvement. In addition to employment status, the desire for work should be routinely assessed.

**Study registration:**

The study was registered in the German Clinical Trials Register (DRKS) (https://www.drks.de/drks_web/navigate.do?navigationId=trial.HTML&TRIAL_ID=DRKS00015801) and under the WHO-Platform “International Clinical Trials Registry Platform” (ICTRP) (https://apps.who.int/trialsearch/Trial2.aspx?TrialID=DRKS00015801) under the registration number DRKS00015801 before the start of recruitment (Registration date: 21.02.2019).

## Background

Mental disorders are associated with negative effects on employment for those affected. It is assumed that patients with a severe mental illness (SMI) are at particular risk of occupational exclusion [1–3]. However, data on the work status of severely mentally ill people are scarce [4].

A literature review from 2004 showed that in Europe, only 10–20% of patients suffering from schizophrenia were employed [5]. An Italian study found that the employment rate in patients with SMI is significantly lower as compared to non-SMI patients (Employed: 6% vs. 39%, *p* < 0.001) [6]. A more recent German study of a large, unselected sample of patients undergoing inpatient psychiatric treatment (*n* = 815) found that only 21% had a permanent employment contract. Many of them did not return to their workplace after being discharged [7]. In Germany, retirement due to disability from mental disorders has risen steadily in recent years. In 2016, 43% of early retirements were related to mental disorders [8]. Furthermore, the number of mentally ill people working in sheltered workshops has been steadily increasing over the last decades. Currently, the proportion of mentally ill people among all those employed in sheltered workshops is 20%. This corresponds to about 60,000 people with severe disabilities due to mental illness [9].

Although Germany offers a range of different vocational rehabilitation programs for mentally ill individuals [10], exclusion from the workforce is still high [11]. Most of the German rehabilitative services follow the principle: “first train–then place”. However, supported employment (SE) initiatives, especially Individual Placement and Support (IPS) programmes [12], are evolving [13]. There is profound evidence in favour of SE [14–16]. In addition to positive effects on employment rates and job retention, positive effects on non-vocational outcomes, such as reduced need for inpatient treatment, reduced psychopathological symptoms, and improved quality of life were reported [17–20]. Therefore, SE is strongly recommended in the German S3 guideline, “Psychosocial therapies for severe mental illness” as the most effective intervention for bringing individuals with SMI back to employment [21]. A requirement to enter an SE-program is the individual’s preference for competitive employment. However, data on the preferences of severely mentally ill people regarding employment are rare and predictors of those preferences have not been studied so far. Therefore, to identify potential target groups for SE-interventions, this study aims to answer the following questions: (1) how strong is the current desire for competitive employment among patients with SMI? (2) What is the current employment situation and how does it relate to the desire to work? (3) What sociodemographic, illness-related and other relevant individual characteristics are associated with a strong preference for work?

## Methods

### Design and setting

This study is a non-interventional, cross-sectional study of patients with SMI conducted within a larger project (Implementation Status of the German Guideline for Psychosocial Interventions for Patients with Severe Mental Illness (IMPPETUS)) [22]. The data were collected in 10 departments of psychiatry and psychotherapy, which provide in- and outpatient psychiatric care for people with mental illnesses in Bavaria (Upper Bavaria and Swabia), including metropolitan catchment areas (Augsburg and Munich), middle-urban regions (Kempten and Memmingen) and rural regions (Donauwoerth, Guenzburg, Kaufbeuren, and Taufkirchen). Recruitment and data collection were carried out from March 2019 to September 2019. The participants were interviewed during their inpatient or day-hospital stay.

### Participants

**Fig. 1:**
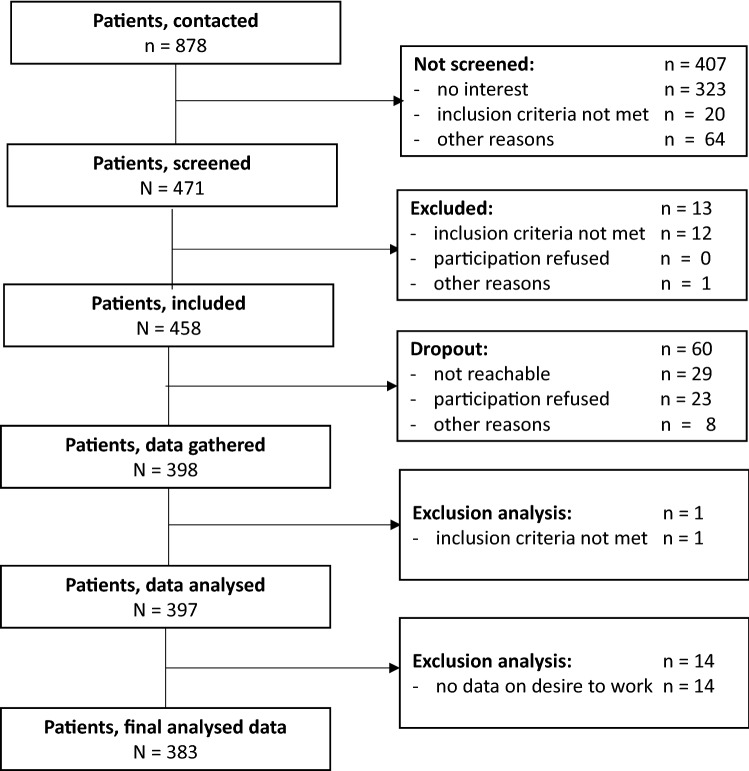
Flow chart of the included patients

A total of 878 patients were initially contacted to participate in the study. Of these, 471 were interested in participating and were screened and 458 patients met the inclusion criteria and agreed to participate. Data were collected from 398 patients. Data could not be gathered from 60 subjects, since they were no longer reachable, had cancelled participation or had other reasons for discontinuation. The analyses were carried out with the data from 383 patients; one patient was excluded from the analyses, because it became apparent afterwards that he did not meet the inclusion criteria (18–65 years). For 14 patients, there were no data available on their desire for work. For nine patients, only the fulfilment of the inclusion criteria was documented, but concrete values are missing for GAF (*n* = 7) or age (*n* = 2). For further details, see Fig. 1.

### Screening and inclusion of participants

Patients were invited by study personnel to participate in the study. Patients who agreed were screened using the "Global Assessment of Functioning" (GAF) [23] and the German version of the "Health of the Nation Outcome Scales" (HoNOS) [24] by trained staff to identify patients with SMI. The HoNOS-D is a 12-item instrument for recording the differentiated severity of a mental illness [25]. The GAF records the general level of functioning taking into account psychological, social and professional aspects of patients with mental disorders [23]. The assessment of the degree of severity of functional impairment is made on a scale of 1–100, with a value of 100–91 reflecting excellent performance and a value of 10–1 reflecting very severely impaired performance. The screening was carried out as soon as possible after admission. The duration of the illness was taken from the medical record or from the information provided by the treating physician.

To identify patients with SMI, the following inclusion criteria were used: (I) patients with schizophrenia, schizotypal and delusional disorders (ICD-10 F2x) and affective disorders (ICD-10 F3x); (II) duration of psychiatric illness ≥ 2 years; and (III) considerable consequences for the activities of daily life and social functioning [21]. The following thresholds were applied to operationalize (III): (1) Global Assessment of Functioning (GAF) [23] from ≤ 60 and (2) Health of the Nation Outcome Scales (HoNOS) [24] score of (a) ≥ 2 on one of the items of the symptomatic problems subscale (scores 6, 7 and 8) and a score of ≥ 2 on each of the four items of the social problems subscale (scores 9, 10, 11, and 12), or (b) a score of ≥ 3 on at least one of these items (9, 10, 11, or 12). Further inclusion criteria were: (IV) 18–65 years, (V) ability to consent, and (VI) German language sufficient to understand the questions. Legal representatives of the patients (if any) were informed about participation in the study, if the patient had consented. Patients who met the inclusion criteria were interviewed by the trained study staff shortly before their discharge.

### Variables

#### Outcome variable

Information on the desire for paid/competitive employment stems from one item of the questionnaire, “Attitudes and Knowledge Regarding Psychosocial Therapies” developed by the authors and available on request. Respondents were asked: “How strong is your current desire for paid employment in the general labour market?” (answer categories: low, medium, or strong). For reasons of evaluation, responses on low and medium categories were collapsed into one group, forming a binary indicator of strong desire for paid/competitive employment (yes/no).

#### Determinants

Socio-demographic data, medical data (e.g., diagnosis of psychiatric disorder, age at first mental problems, and presence of a chronic physical illness), and employment-related data of the patients were assessed with items taken from the “Measure of participation and social inclusion for use in people with a chronic mental disorder” (F-INK) [26] and the “Client sociodemographic and service receipt inventory” (CSSRI) [27]. The F-INK is a modular questionnaire with nine modules, which covers the key dimensions of social inclusion. The CSSRI is a semi-structured interview to collect social and demographic data, detailed information on treatment, physician visits, and the use of social and health services. If the respondent or one of his/her parents was born abroad, the respondent was categorized as an immigrant. Patients living alone were distinguished from those not currently alone (with partner, children, parents, siblings, and other relatives, with friends or others).

Questions regarding the knowledge about SE and additional training at the workplace (e.g., social skills training, job-related training, and cognitive training) stem from items of the Questionnaire “Attitudes and Knowledge Regarding Psychosocial Therapies” (yes/no). The current ability to work compared to the best ability to work ever achieved was surveyed with WAI 1 [28]. WAI 1 consists of the single item, “Assume that your work ability at its best has a value of 10 points. How many points would you give your current work ability?” (0 = Completely unable to work, 10 = Work ability at its best).

### Analyses

Absolute and relative frequencies, means and SDs were calculated as descriptive statistics. Group comparisons between strong and non-strong desire for paid employment were calculated using Chi-square tests for categorical variables and Wilcoxon two-sample tests for continuous variables. The relationship of determinants (e.g., age, gender, diagnosis) with a strong preference for paid employment was analysed using hierarchical binary logistic regression models. The analyses followed a blockwise modelling approach. Model 1 assessed the association between sociodemographic factors and a strong preference for competitive employment. Model 2 additionally quantified the impact of illness-related factors. Finally, model 3 additionally assessed the contribution of subjective work-related factors. The blockwise modelling allowed us to quantify the extent to which the effect of single determinants increased/decreased by adding further covariables. Wald *χ*^2^ statistics are used to test the significance of predictor variables in the models. Data were missing for < 10% of all covariates and handled by casewise deletion, since sensitivity analysis revealed no indication of systematic biases due to missing data. The corresponding sample sizes for each subgroup comparison are reported in the tables. All statistical analyses were performed using Stata 15.1 MP (StataCorp LP, College Station, TX) and IBM SPSS Statistics for Windows, version 24 (IBM Corp., Armonk. NY).

## Results

In total, 383 patients with a mean age of 42.7 years (SD 13.1 years) were included in the analysis. More than half of the respondents were women (*n* = 215, 56.1%). Similarly, more than half of participants were single (*n* = 215, 56.2%); 158 patients (41.9%) lived alone. Seventy patients (18.4%) stated that they or a parent were born in another country. The majority of patients had a diagnosis of depression (*n* = 227, 59.3%), almost one-third of patients had a schizophrenia spectrum disorder (*n* = 118, 30.8%), and almost one-tenth had a bipolar disorder (*n* = 38, 9.9%). The psychosocial impairments were considerable (GAF, means (SD): 42.3 (9.8)) corresponding to severe disease impairment according to linkage analyses [29]. Half of the respondents reported comorbid physical diseases (*n* = 192, 50.4%). Work ability (WAI 1) was given an average rating of 4.0 (SD 2.7). Further characteristics of the study participants are shown in Table 1.

**Table 1:** Sociodemographic, clinical and other characteristics of study participants according to their preference for competitive work (*n* = 383)

	All patients	Strong desire for competitive work		Value Wilcoxon two-sample test/Chi-square test (Pearson) (df)
	*n* = 383	No *n* = 154 (40.2)	Yes *n* = 229 (59.8)	
Age (years), mean (SD) (*n* = 381)	42.7 (13.1)	44.3 (13.2)	41.6 (12.9)	*z* = 1.97*
Sex, *n* (%)				*χ*^2^ = 6.95 (1)**
Male	168 (43.9)	55 (35.7)	113 (49.3)	
Female	215 (56.1)	99 (64.3)	116 (50.7)	
Education, *n* (%) (*n* = 382)				*χ*^2^ = 2.43 (2)
Low	146 (38.2)	60 (39.0)	86 (37.7)	
Medium	111 (29.1)	50 (32.5)	61 (26.8)	
High	125 (32.7)	44 (28.5)	81 (35.5)	
Marital status, *n* (%)				*χ*^2^ = 0.38 (2)
Single	215 (56.2)	84 (54.5)	131 (57.2)	
Married/registered partnership	89 (23.2)	36 (23.4)	53 (23.1)	
Divorced/widowed/separated	79 (20.6)	34 (22.1)	45 (19.7)	
Living situation^a^, *n* (%) (*n* = 377)				*χ*^2^ = 0.24 (2)
Alone	158 (41.9)	66 (43.4)	92 (40.9)	
Not alone	219 (58.1)	86 (56.6)	133 (59.1)	
Immigration background, *n* (%) (*n* = 381)				*χ*^2^ = 0.61 (1)
No	311 (81.6)	122 (79.7)	189 (82.9)	
Yes	70 (18.4)	31 (20.3)	39 (17.1)	
Mental disorder, *n* (%)				*χ*^2^ = 5.83 (2)
F2x	118 (30.8)	56 (36.4)	62 (27.1)	
F32, F33	227 (59.3)	88 (57.1)	139 (60.7)	
F30, F31	38 (9.9)	10 (6.5)	28 (12.2)	
GAF, means (SD) (*n* = 376)	42.3 (9.8)	40.6 (10.1)	43.4 (9.4)	*z* = − 2.74**
HoNOS-D, mean (SD)	21.8 (6.0)	22.6 (5.8)	21.2 (6.0)	*z* = 2.53*
Age at first mental problems (years), mean (SD) (*n* = 359)	26.8 (13.0)	27.2 (12.7)	26.6 (13.2)	*z* = 0.44
Physical illness, *n* (%) (*n* = 381)				*χ*^2^ = 12.47 (1)***
Yes	192 (50.4)	94 (61.4)	98 (43.0)	
No	189 (49.6)	59 (38.6)	130 (57.0)	
Knowledge of vocational rehabilitation measures, *n* (%) (*n* = 382)				
Supported employment, Yes	115 (30.1)	41 (26.6)	74 (32.5)	*χ*^2^ = 1.49 (1)
Additional training^b^, Yes	146 (38.2)	52 (33.8)	94 (41.2)	*χ*^2^ = 2.17 (1)
Work ability, mean (SD) (*n* = 379)	4.0 (2.7)	3.0 (2.3)	4.7 (2.8)	*z* = − 5.88***

Subsample sizes vary due to missing information. Corresponding sizes for variables with missing data are given in brackets

*GAF* Global assessment of functioning, *HoNOS-D* German Version of the Health of the Nation Outcome Scales, *SD* standard deviation, *F2x* Schizophrenia, schizotypic and delusional disorders, *F32, F33* Depressive disorders, *F30, F31* Mania and bipolar disorder

*p < 0.05

**p < 0.01

***p < 0.001

^a^In terms of a social component

^b^e.g., social skills training, job-related training, cognitive training

### Employment status and desire for work

**Fig. 2:**
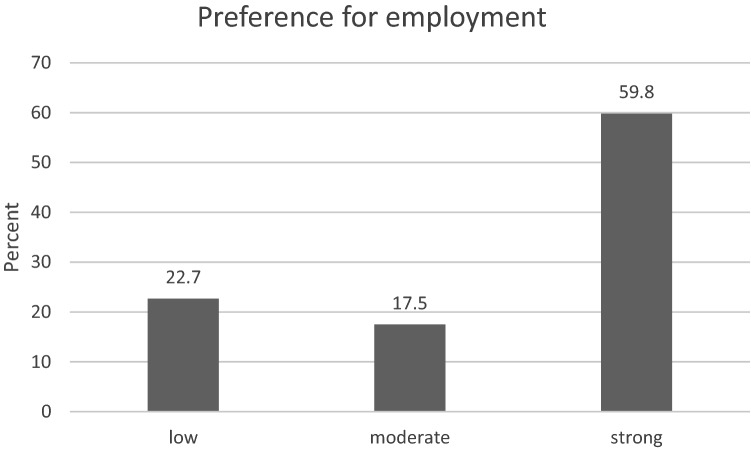
Proportion of respondents with a low, moderate and strong preference for employment in the general labour market

The majority of patients indicated a strong preference for competitive employment (*n* = 229, 59.8%). Another 87 patients (22.7%) stated a low preference, and 67 patients (17.5%) a moderate preference (Fig. 2).

Employment information was available for 348 patients. 97 patients (27.9%) were competitively employed. 90 patients (25.9%) surveyed were without employment and seven patients (2.0%) were marginally employed. In addition, 17 patients (4.9%) were engaged in sheltered activities, 81 patients (23.3%) had retired for health reasons and 24 patients (6.9%) were enrolled in school, vocational training or university. Finally, 32 patients (9.2%) stated that they were drawing retirement pensions, looking after children or running a household (Table 2).

**Table 2:** Description of the current work situation (*n* = 348)

	All patients	Strong desire to work in general labour market						Value Chi-square test (Pearson) (df)
	*n* = 348	No *n* = 146 (42.0%)			Yes *n* = 202 (58.0%)			
	*n* (%)^2^	*N*	%^2^	%^3^	*N*	%^2^	%^3^	
Vocational situation, *n* (%)								
Employed, general labour market	97 (27.9)	20	13.7	20.6	77	38.1	79.4	22.94 (1)***
Sheltered employed	17 (4.9)	8	5.5	47.1	9	4.4	52.9	0.26 (1)
Marginally employed	7 (2.0)	3	2.0	42.9	4	2.0	57.1	–^1^
Unemployed	90 (25.9)	31	21.2	34.4	59	29.2	65.6	2.21 (1)
Early retired	81 (23.3)	55	37.7	67.9	26	12.9	32.1	30.73 (1)***
In school, education, or study	24 (6.9)	9	6.2	37.5	15	7.4	62.5	–^1^
Parental leave, retirement pension or housekeeping	32 (9.2)	20	13.7	62.5	12	6.0	37.5	–^1^

*p < 0.05

**p < 0.01

***p < 0.001

^1^Differences were not examined, because the number in individual cells was too small or irrelevant in terms of content

^2^Relative frequencies over column

^3^Relative frequencies over rows

Patients competitively employed were significantly more likely to have a strong preference for employment compared to others (79.4% vs. 20.6%, *p* < 0.001). Patients in early retirement were significantly less likely than others to indicate a strong preference for work (32.1% vs. 67.9%, *p* < 0.001). There were no significant differences between the two groups in terms of unemployment and sheltered employment (Table 2).

With regard to the group of patients with strong employment preference, slightly more than one-third of patients surveyed were competitively employed (*n* = 77, 38.1%). Nevertheless, a considerable proportion of those surveyed were unemployed (*n* = 59, 29.2%), in sheltered employment (*n* = 9, 4.4%) or retired due to health problems (*n* = 26, 12.9%) expressed a strong desire for a job in the general labour market. This also applies to some of the respondents who were in marginal employment, in training or at home (Table 2).

Looking explicitly at the group of patients without a current job (e.g., those unemployed), two-thirds (*n* = 59, 65.6%) expressed a strong desire to work in the general labour market. Accordingly, only one-third of the currently unemployed did not do so (Table 2).

### Predictors of a strong preference for competitive employment

Table 1 provides an overview of the differences between the patients with strong preference for competitive employment compared to those without a strong preference. With regard to sociodemographic variables, the results show that patients with strong preference are younger (*p* = 0.048) and more likely to be men (*p* = 0.008). No differences were observed with regard to education, marital status, living situation, and immigration. In terms of illness, the results indicate greater psychosocial impairment (GAF, *p* = 0.006), a higher severity of mental disorder (HoNOS-D, *p* = 0.011) and a higher proportion of additional chronic physical illnesses (*p* < 0.001) in the group containing individuals with a low or moderate desire to work. The specific diagnosis of mental illness and the age at which the first mental problems occurred did not differ significantly between the two groups. Patients with a strong desire for work rated their current ability to work significantly better (*p* < 0.001).

For binary logistic regression analysis (*n* = 342 cases with complete data), three models are shown in Table 3, investigating sociodemographic, illness-related, and work-related variables with a strong desire for paid employment. Model 1 revealed that male gender increased the likelihood of a strong preference for competitive employment (OR = 1.81 [95% CI 1.17–2.80], *p* = 0.007). The comparison of model 1 with models 2 and 3 showed that this association became insignificant when additional illness- and work-related factors were considered. Model 2 indicated that chronic physical illness was significantly associated with a preference for competitive employment, adjusting for sociodemographics. The presence of a comorbid chronic physical illness compared to a status free of chronic physical illness reduced the odds ratio of respondents stating a strong preference by almost 50% (OR = 0.48 [95% CI 0.30–0.79], *p* = 0.004). In addition, when work-related predictors were included in model 3, the negative association between comorbid chronic physical diseases and a strong desire for paid employment was still significant (OR = 0.58 [95% CI 0.35–0.97], *p* = 0.039). Model 3 also showed an increase of the odds ratio of a strong preference for patients with bipolar disorder as compared to patients with schizophrenic disorder (OR = 3.53 [95% CI 1.30–9.60], *p* = 0.013). In model 3, knowledge of vocational rehabilitation measures had no significant effect, but the likelihood of a preference for competitive employment increased significantly with a higher subjective work ability (OR = 1.25 [95% CI 1.14–1.38], *p* < 0.001).

**Table 3:** Social, health and work related determinants for strong preference for competitive work in patients with SMI (*n* = 342): results of hierarchical binary logistic regression analyses

Indicator variable	Category	Model 1 (*n* = 373)			Model 2 (*n* = 344)			Model 3 (*n* = 342)		
		OR	95% CI		OR	95% CI		OR	95% CI	
Gender	Female	Ref								
	Male	1.81**	1.17	2.80	1.55	0.96	2.49	1.53	0.93	2.50
Age		0.98	0.96	1.00	0.99	0.96	1.01	0.99	0.96	1.02
Education	Low	Ref	*χ*^2^ = 1.96			*χ*^2^ = 2.44			*χ*^2^ = 2.07	
	Medium	0.97	0.58	1.63	0.82	0.47	1.45	0.89	0.49	1.63
	High	1.38	0.82	2.32	1.31	0.74	2.30	1.37	0.76	2.48
Marital status	Single	Ref	*χ*^2^ = 1.65			*χ*^2^ = 0.12			*χ*^2^ = 0.64	
	Married	1.43	0.74	2.75	1.08	0.51	2.32	1.33	0.60	2.92
	Divorced/widowed/separated	1.44	0.76	2.73	1.13	0.57	2.24	1.27	0.63	2.57
Living situation^a^	Alone	Ref								
	Not alone	1.06	0.65	1.74	1.03	0.60	1.75	0.95	0.54	1.67
Migration background	No	Ref								
	Yes	0.76	0.44	1.32	0.96	0.52	1.79	0.80	0.41	1.55
Mental disorder	F2x				Ref	*χ*^2^ = 5.56			***χ***^**2**^ = 6.37*	
	F32, F33				1.24	0.72	2.14	1.55	0.88	2.75
	F30, F31				2.99*	1.20	7.46	3.53*	1.30	9.60
Age at first problems					1.00	0.98	1.03	1.00	0.97	1.02
GAF					1.02	1.00	1.05	1.01	0.99	1.04
HoNOS-D					0.99	0.94	1.03	1.01	0.96	1.05
Physical illness	No				Ref					
	Yes				0.48**	0.30	0.79	0.58*	0.35	0.97
Knowledge, SE	No							Ref		
	Yes							1.50	0.85	2.64
Knowledge, additionnal training	No							Ref		
	Yes							1.05	0.62	1.79
Work ability								1.25***	1.14	1.38
	R^2^ (McFadden)	0.028			0.071			0.123		

*χ*^2^ indicating the significance of the predictor variable in the models by wald test

*SE* Supported Employment, *GAF* Global assessment of functioning, *HoNOS-D* German Version of the Health of the Nation Outcome Scales, *F2x* Schizophrenia, schizotypic and delusional disorders, *F32, F33* Depressive disorders, *F30, F31* Mania and bipolar disorder, *OR* Odds ratio, *CI* Confidence interval

Boldface indicates statistical significance: *p < 0.05, **p < 0.01, ***p < 0.001

^a^In terms of a social component

## Discussion

### Employment status and desire for work

Our data confirm a substantial exclusion of individuals suffering SMI from the workforce. Just over half of the patients surveyed reported being unemployed, retired early due to mental illness, or in sheltered employment. Only one-quarter (27%) of the SMI patients is engaged in competitive employment. However, a desire for competitive employment is highly ranked with more than half of the SMI patients having a strong preference for employment in the general labour market. Of patients in the subgroup, currently unemployed, 65% endorsed a strong preference for competitive employment. This is of utmost importance, since a high interest in work increases the likelihood of future employment [30, 31]. On the other hand, lack of motivation was found to be one of the most significant barriers to re-entering workforce [32, 33].

Few studies have investigated the desire for competitive work. Westscott et al. (2015) showed that 34% of community-based patients with a diagnosis of schizophrenia or schizoaffective disorder (*n* = 255) were currently employed and an additional 51% were interested in employment. In total, 85% were already participating in, or interested in, employment [34]. In another community-based sample of persons with SMI (*n* = 166), 30% of the participants expressed no interest in getting a job [35]. In a recent study examining a German sample of inpatients comparable to our sample, 71% of the participants stated a short- or medium-term interest in work or training/study [36]. In contrast, the proportion of inpatients with a strong desire to work was estimated to be significantly lower in a Belgian study. A set of multiple-response questions asked patients to indicate their short- and long-term vocational goals, including competitive employment. In the short term, 35.5% of the patients preferred competitive employment and 21.8% preferred voluntary work. A similar picture emerged when considering long term goals with 44.6% preferring competitive employment and 14.6% opting for voluntary employment. Patients from long-term wards were included in this sample [37]. Mueser et al. examined 528 patients with schizophrenia who had had a psychiatric hospitalisation or symptom exacerbation in the past 3 months. Among patients who were not competitively employed, 61% reported an interest in working [30]. Although the results are heterogenous, most of the studies reported quite a substantial number of patients with SMI having a strong desire for work. Differences may be attributed to variations in patient samples and the wording of the questions asked to inquire about desire for work.

Our findings point to the need for targeted job-related interventions among patients with SMI. Two-thirds of individuals who are currently unemployed expressed a high desire for work and, therefore, constitute an obvious target group for SE interventions. The basic principles of SE and Individual Placement and Support include a focus on competitive employment based on user preference. The approach was tailored for severely mentally ill people and has been proven effective in numerous studies [14]. Users are supported through individualised and long-term on-the-job support (job coaching), while mental health and employment services are closely integrated [12]. The need for appropriate support is not currently being met in Germany. A recent study showed that 71% of patients not working or enrolled in an educational program indicated that "work" was a relevant topic. Specifically, 63% would like vocational support; of these, 84% would participate in job coaching. Young adults (77%) and first-time sufferers (73%) indicated an even higher need for support [36].

Interventions are also required for other groups who would like to make the step to a competitive employment such as people who have retired due to health problems or those who are in sheltered employment with high desire to do more. In fact, almost one-third of the respondents in early retirement (32%) and more than half of the respondents in sheltered employment (53%) affirmed a strong desire for competitive work. Similarly, patients who have a rather low interest in work or who are ambivalent should receive interventions to increase interest and motivation. Attitudes of mental health professionals towards their patients and the social environment are important, as interest in work can be altered not only by one’s own expectations, but also by the expectations of others. Healthcare professionals, in particular, can reinforce negative expectations if they do not consistently address work-life and return-to-work issues [34]. It is beneficial to promote patients' confidence and self-esteem [38].

### Predictors of the desire for work

Three predictors of a high desire to work have emerged as a result of the present work. Among the disease-related aspects, an additional physical illness seems to have a major influence on the desire to work. The presence of a comorbid chronic physical illness reduced the likelihood that respondents indicated a strong preference by almost 50%. In terms of diagnosis, individuals suffering from bipolar disorder were more likely to express a strong desire to work than patients with a schizophrenia disorder. Patients' self-rated work ability is associated with a stronger desire to work. However, the WAI 1 score level (without strong desire 3.0 [SD: 2.3] vs. with strong desire 4.7 [SD: 2.8]) is relatively low in both groups. To our knowledge, this is the first study to examine predictors of desire for competitive work among patients with SMI.

It is possible that the predictors differ from those on the employment status of people with SMI. However, there is also evidence for comparable findings. A study of SE programs in unemployed individuals showed that the presence of a comorbid physical condition was predictive of lower rates of competitive employment, fewer hours worked, and lower wages earned over the 2-year follow-up period [39]. A negative association between somatic comorbidity and employment was also observed in other studies [40, 41]. Other predictors are also discussed in connection with employment in patients with SMI. Some of the findings are considered contradictory. In addition to functioning, sociodemographic factors and work history [5, 30, 42, 43], for example, a connection between stigma and discrimination in the workplace and employment [44] and between the type of vocational rehabilitation measure and work [45] is also described. The low values of the explained variance in our models suggest that further predictors would have an influence on the desire to work.

### Limitations

Although a large sample of SMI patients was investigated, selection bias cannot be ruled out. This holds true in terms of recruitment strategies, as well as in response to the invitation to the study. The results refer only to patients with SMI in the Bavarian region of Germany. We investigated desire for work as a relevant but simple real-life question. It is possible that related, more comprehensive constructs and instruments such as return to work self-efficacy [46] or motivational aspects can provide deeper insights [47]. The data were collected in a cross-sectional study so that co-variations cannot be interpreted causally.

### Conclusions

Employment is considered to be an important determinant of health and a milestone in the recovery process of people with SMI [48]. Our findings show that the desire for competitive work is strong among more than half of patients with SMI. Likewise, exclusion of these patients from the workforce is high. Among the unemployed, two-thirds express a strong desire for work. These individuals comprise a clear target group for SE interventions. There is much room for improvement, because the need for workplace interventions is not adequately met. Mental health professionals must routinely assess both employment status and desire for work. Based on the assumption that the desire for work changes over time and can be modified [49], motivation to work should be strengthened throughout the course of treatment. Attitudes of mental health professionals are relevant, and issues of work-life balance should be addressed throughout the course of treatment. The data also underline the relevance of somatic comorbidity for work-related outcomes. In programmes intended to strengthen labour market integration, it is important to consider the physical health status of patients.

## Data Availability

The data sets used and analysed in the presented analyses are available from the corresponding author on reasonable request.
